# Journal De La Physiologie De L’Homme Et Des Animaux

**Published:** 1858-05

**Authors:** 


JOURNAL DE LA PHYSIOLOGIE DE L'HOMME ET DES ANIMAUX.—We take great pleasure in announcing the receipt of the first number of this journal, edited by Dr. E. Brown-Séquard, which contains 216 pages, more than three-fourths of which are devoted to original articles. These are thirteen in number, six of which are contributed by Dr. Brown-Séquard himself. An article on Digestion, from the pen of Dr. Francis G. Smith, of this city, which was published in the *Medical Examiner* more than a year since, has been translated, and finds a place in the original matter. Also an article on the transformation of starch into glucose in the stomach, by Drs. F. G. Smith and Brown-Séquard. We hope to be able to give selections from, or translations of, the most interesting articles as they appear from time to time.

